# YTHDF2 Inhibits the Migration and Invasion of Lung Adenocarcinoma by Negatively Regulating the FAM83D-TGFβ1-SMAD2/3 Pathway

**DOI:** 10.3389/fonc.2022.763341

**Published:** 2022-02-02

**Authors:** Teng Zhao, Mingchao Wang, Xin Zhao, Shuang Weng, Kun Qian, Kejian Shi, Yanfei Gu, Wantao Ying, Xiaohong Qian, Yi Zhang

**Affiliations:** ^1^ Department of Thoracic Surgery, Xuanwu Hospital, Capital Medical University, Beijing, China; ^2^ State Key Laboratory of Proteomics, Beijing Proteome Research Center, National Center for Protein Sciences (Beijing), Beijing Institute of Lifeomics, Beijing, China; ^3^ Department of Oncology, United Family New Hope Oncology Center, Beijing, China

**Keywords:** NSCLC, N6-methyladenosine, YTHDF2, FAM83D, TGFβ1

## Abstract

**Objective:**

YTH domain family 2 (YTHDF2) is an important N6-methyladenosine (m6A) reader, but its role in lung adenocarcinoma remains elusive. This study assessed its function in lung adenocarcinoma.

**Methods:**

YTHDF2 expression in lung adenocarcinoma was explored using public databases, such as The Cancer Genome Atlas (TCGA) and the Clinical Proteomic Tumour Analysis Consortium (CPTAC). The effect of YTHDF2 on a lung adenocarcinoma cell line was explored by performing cytological and molecular experiments. Molecules downstream of YTHDF2 were identified using proteomics, and the related pathways were verified through cytological and molecular biology experiments.

**Results:**

YTHDF2 expression was upregulated in lung adenocarcinoma, and patients with high YTHDF2 expression experienced prolonged overall survival. In two lung cancer cell lines, YTHDF2 knockdown inhibited proliferation but promoted migration, invasion, and the epithelial-mesenchymal transition. The proteomic analysis identified 142 molecules downstream of YTHDF2, and 11 were closely related to survival. Further experiments revealed that YTHDF2 inhibited expression of the family with sequence similarity 83D (FAM83D)-TGFβ1-SMAD2/3 pathway components. This study is the first to show that YTHDF2 regulated the downstream TGFβ1-SMAD2/3 pathway through FAM83D in lung adenocarcinoma.

**Conclusion:**

YTHDF2 inhibits the migration and invasion of lung adenocarcinoma cells by regulating the FAM83D-TGFβ1-pSMAD2/3 pathway, which may play an important role in lung cancer metastasis.

**Graphical Abstract:**
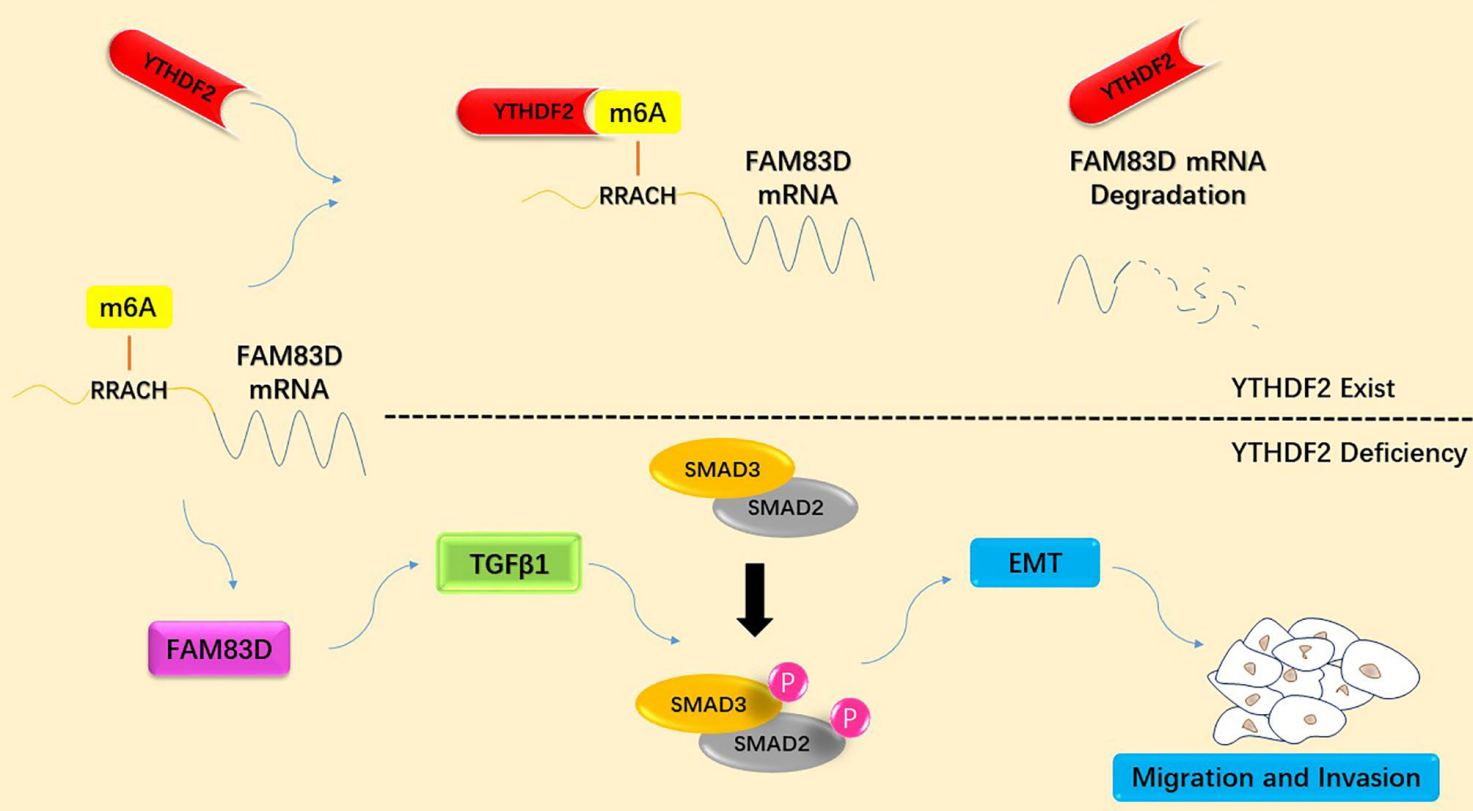
Schematic diagram summarizing the molecular mechanism by which YTHDF2 inhibits the migration and invasion of lung adenocarcinoma cells by negatively regulating the FAM83D-TGFβ1-SMAD2/3 pathway.

## Introduction

Lung cancer is one of the most common malignant tumours worldwide and causes a large number of deaths every year. Moreover, the economic burden caused by lung cancer has become one of the most serious public health problems in recent years. Although the diagnosis and management of lung cancer has improved substantially in recent years, the overall five-year survival rate of patients with lung cancer is still less than 20% (1). Non-small cell lung cancer (NSCLC) consists of many subtypes, such as lung squamous cell lung carcinoma, lung adenocarcinoma, large cell carcinoma and sarcoma. Currently, genetic mutation-oriented individualized therapy has become the standard treatment for NSCLC, especially lung adenocarcinoma.

Methylation of the N6 site on adenosine (N6-methyladenosine, m6A) is one of the most common RNA modifications in eukaryotes. The m6A modification has been shown to be a reversible process involving many molecules, such as methyltransferases (writers), demethylases (erasers) and methylated reading proteins (readers) (2). The m6A modification plays an important role in the development of many tumours, including lung adenocarcinoma (3).

The YTH domain family (YTHDF) is a series of important reader proteins for m6A methylation. Five major proteins in this family have been identified: YTHDC1, YTHDC2, YTHDF1, YTHDF2 and YTHDF3 (4). Different functions related to tumour and other diseases of these proteins have been discovered in recent years (5–14). However, the role of YTHDF2 in lung adenocarcinoma remains unclear. In this study, through the comprehensive analysis of multiple public databases, we identified abnormal YTHDF2 expression in lung adenocarcinoma. Moreover, we found for the first time that YTHDF2 could inhibit the migration and invasion of lung adenocarcinoma cells by regulating the epithelial-mesenchymal transition (EMT) through the family with sequence similarity 83D (FAM83D)-TGFβ1-SMAD2/3 pathway.

## Materials and Methods

### Public Datasets

We explored YTHDF2 expression in patients with lung adenocarcinoma using The Cancer Genome Atlas (TCGA) and Clinical Proteomic Tumour Analysis Consortium (CPTAC) (15) databases. Patient information, clinical outcomes, and mRNA levels in normal and cancer tissues in TCGA lung adenocarcinoma (TCGA-LUAD) dataset were obtained from the UALCAN browser (16) (http://ualcan.path.uab.edu/analysis.htm) and UCSC XENA browser (17) (https://xenabrowser.net). In our study, patients were grouped by sex, stage, race and age. Protein expression data in the CPTAC database were obtained from the UALCAN browser. The prediction of m6A modification site on mRNA was performed by SRAMP prediction server (18).

### Validation With the Chinese Human Proteome Project (CNHPP) Database

The lung cancer proteomics data in the CNHPP database (19) were used to verify the expression of YTHDF2 in the Asian population. YTHDF2 protein expression levels in 103 pairs of lung adenocarcinoma tissues and adjacent normal tissues were determined in this study.

### Cell Culture

Two different lung adenocarcinoma cell lines, A549 and H1299, were selected for the study. Cell lines were obtained from Capital Medical University. All cells we used were proven to be free from bacteria, fungi and mycoplasma by PCR. Dulbecco’s modified Eagle’s medium (DMEM) (Corning, USA) containing 10% FBS (Gibco, USA) was used to culture cells. Penicillin and streptomycin (Gibco, USA) were added to the medium at suitable concentrations to prevent cellular contamination. Cells were cultured in an incubator at 37°C with 5% CO_2_.

### Cell Transfections

Transfection was performed when the cell confluence reached 60-70%. The small interfering RNA (siRNA) was designed to knockdown the target gene and synthesized by Sangon Biotech Co., Ltd. (Shanghai). Thermo Lipofectamine™ RNAiMAX transfection reagent (Thermo Fisher Scientific, USA) was used to transfect the siRNA into cells. Plasmids were designed to overexpress the target gene and synthesized by Sangon Biotech Co., Ltd. (Shanghai). Thermo Lipofectamine™ 3000 Reagent (Thermo Fisher Scientific, USA) was used to transfect plasmids into cells. Antibiotic-free medium was used after transfection to prevent cell injury. RNA was extracted 48 hours after transfection, and protein extraction was performed 72 hours after transfection to ensure knockdown or overexpression.

### Protein Extraction

The samples (tissue or cells) were washed with PBS (Gibco, USA) to remove unnecessary components, such as blood and culture medium. The washed samples were lysed with T-PER™ tissue protein extraction reagent (Thermo Fisher Scientific, USA) *via* an ultrasonic cell disruptor, in which tissues were cut into small pieces, and cells were blown into suspension. Protease inhibitors and phosphatase inhibitors were added to the extraction reagent to inhibit protein degradation and protein dephosphorylation. Then, samples were centrifuged at 14,000 g for 5 min to remove the debris. The protein concentration was measured with Coomassie Plus™ Protein Assay Reagent (Thermo Fisher Scientific, USA), and the protein solution was stored at -80°C.

### RNA Extraction

TRIzol reagent from Invitrogen (Thermo Fisher Scientific, USA) was used to extract total RNA from samples, and isopropanol was used to separate RNA from the reaction system. The RNA was washed twice with 75% ethanol, and the concentration of RNA was measured with a Thermo Scientific NanoDrop™ spectrophotometer. Optical density (OD) values were measured at different wavelengths, and samples with OD260/280 values greater than 2 were considered qualified.

### Cell Proliferation

Cell proliferation was evaluated using MTT assay and colony formation assay. In the MTT assay, normal cancer cell lines and siRNA-transfected cell lines were cultured in 96-well plates at the same cell density. The MTT assay was performed every 8 hours, and the OD value at 458 nm was measured using a microplate spectrophotometer. The growth curve was drawn according to the OD value. In the colony formation assay, normal cancer cell lines and siRNA-transfected cell lines were cultured at the same cell density, immobilized with methanol and stained with crystal violet after 72 hours.

### Cell Migration

A wound healing assay was performed to evaluate cell migration. Normal cancer cell lines and siRNA-transfected cell lines were cultured in 6-well plates at the same cell density, and plates were scratched with a pipette tip after cells reached confluence. Then, the cells were cultured in DMEM without FBS to prevent cell proliferation. The width of the scratch was measured under a microscope every 8 hours.

### Cell Invasion

Transwell assays were performed to evaluate cell invasion. Normal cancer cell lines and siRNA-transfected cell lines were cultured in FBS-free DMEM in a Transwell chamber (Corning, USA) coated with Matrigel matrix. Twenty-four hours later, Transwell chambers were placed in 24-well plates containing DMEM supplemented with 15% FBS. Forty-eight hours later, the Transwell chambers were treated with methanol and crystal violet, and the number of cells and colonies on the lower side of the Transwell chamber were observed.

### Flow Cytometry

The flow cytometry analysis was performed with a *BD* FACS *Verse* flow cytometer to detect the number of apoptotic cells and cell cycle status of YTHDF2-silenced cells and normal lung adenocarcinoma cell lines. In the apoptosis assay, an Annexin V-FITC/propidium iodide (PI) kit was used to stain cells at room temperature for 30 min, and then the cells were resuspended in PBS. The cell detection rate was 300 per second. In the cell cycle assay, cells were fixed with ethanol at 4°C for 2 hours, incubated with an RNase solution at 37°C for 30 minutes, and PI staining was performed at room temperature for 30 min. The data were analysed using FlowJo v10.8 software.

### qRT–PCR

Quantitative reverse transcription PCR (qRT–PCR) was used to evaluate mRNA levels. The primers in our study were synthesized by Sangon Biotech (Shanghai) Co., Ltd. Details of the primer sequences are provided in the **Supplementary Materials**. The One-Step RT–PCR Kit (Analytik Jena, Germany) was used in our study with the Analytik Jena qTOWER 2.2 thermal cycler. Actin was used as the internal reference. The fluorescence intensity of SYBR Green was captured in real time. The cycle threshold (CT) value was used to calculate relative mRNA expression levels.

### Western Blotting (WB)

The total protein concentration was detected using Coomassie Plus™ Protein Assay Reagent. SDS–PAGE was used for protein electrophoresis. Proteins were resolved on a 5% concentrating gel at 80 V for 30 min, followed by a 12% separation gel at 120 V for 100 min. Proteins were transferred to a 0.45-nm PVDF membrane using a Bio-Rad Trans-Blot SD Semi-Dry Transfer Cell. The PVDF membrane was incubated with the primary antibody for 16 hours at 4°C and then incubated with secondary antibody for 2 hours at room temperature. Chemiluminescence was performed with a horseradish peroxidase (HRP) substrate, and the signal was captured with a GE ImageQuant™ LAS 500 instrument. The primary and secondary antibodies used in the study are listed in the **Supplementary Materials**.

### RNA Binding Protein Immunoprecipitation (RIP)

A549 cells were collected with trypsin (2*15-cm plates), pelleted by centrifugation for 5 min at 1,000 g and washed once with cold PBS. The cell pellet was resuspended and incubated on ice for 20 min with lysis buffer 1 (0.5% SDS in PBS supplemented with a 1:100 dilution of a protease inhibitor cocktail and 400 U/ml RNase inhibitor; one plate was incubated with 250 µl of lysis buffer 1). Then, the lysate was incubated on ice for 20 min with lysis buffer 2 (0.2% Triton 100 in PBS supplemented with a 1:100 dilution of a protease inhibitor cocktail and 400 U/ml RNase inhibitor; one plate was incubated with 1,000 µl of lysis buffer 2). After the incubation, the lysate was centrifuged at 14,000 g for 20 min. The cell lysate was rotated continuously with the anti-YTHDF2 antibody at 4°C for 16 hours, and then washed Protein A/G magnetic beads (Biomake, 100 µl to each sample) were added to the mixture. After 4 hours of incubation, the magnetic beads were isolated and mixed with Invitrogen TRIzol reagent; the tube was flicked several times to mix the contents, and RNA was extracted with chloroform. Isopropanol was used to precipitate RNA, while ethanol was used to wash the precipitant. RNA samples were obtained from the purified precipitant and prepared for subsequent experiments, such as qRT–PCR.

### Peptide Preparation

Filter-aided sample preparation (FASP) was used to prepare peptides, the protein was cut into peptides with trypsin, and the concentration of peptide was measured with a Thermo Scientific™ NanoDrop™ spectrophotometer. The product was lyophilized at a low temperature and stored at -80°C.

### LC–MS/MS

The LC–MS/MS detection system consisted of a nanoflow high-performance liquid chromatography (HPLC) instrument (Easy nLC1000 System, Thermo Fisher Scientific, USA) coupled to a Q Exactive HF mass spectrometer (Thermo Fisher Scientific, USA). A self-packing C18 reverse-phase column (150 µm×30 cm, 1.9 μm) was used for peptide separation at 60°C. The flow rate was 500 nL/min over a 135-min gradient (0-13 min, 6-10% B; 13-99 min, 10-23% B; 99-120 min, 23-33% B; 120-123 min, 33-90% B; and 123-135 min, 90% B). A full mass spectrometry survey scan ranged from 375 to 1,400 m/z.

The DDA raw data files were searched against the human UniProt database (20140922, added with sequences of iRT peptides) with MaxQuant (version 1.5.3.8) using the default settings. The false discovery rate (FDR) was set to 0.01 for both peptides and proteins.

### Data Processing and Statistical Analysis

Data from public datasets are presented as the means ± standard deviations (SD). Student’s t test and z test were used to compare differences between two groups with SPSS 22.0 statistical software (IBM Inc. Chicago, USA). The cut-off value was calculated by performing a receiver operating characteristic curve (ROC) analysis using MedCalc v18.5 software, and a Kaplan–Meier survival analysis was conducted to explore the differences in the prognosis. Flow cytometry data were analysed using FlowJo v10 software (BD Inc., New York, USA). WB and PCR were repeated at least 3 times, and the greyscale values of the bands were measured using ImageJ v1.8.0 software (NIH, USA). The data were analysed using SPSS 22.0 Statistical Software, and the bar chart was drawn with SigmaPlot v12.5 software (Systat Software Inc, California, USA). A P value <0.05 was considered to indicate a statistically significant difference.

## Results

### YTHDF2 Is Expressed at High Levels in Lung Adenocarcinoma, and Patients With Higher YTHDF2 Expression Have a Better Prognosis

**Figure 1:**
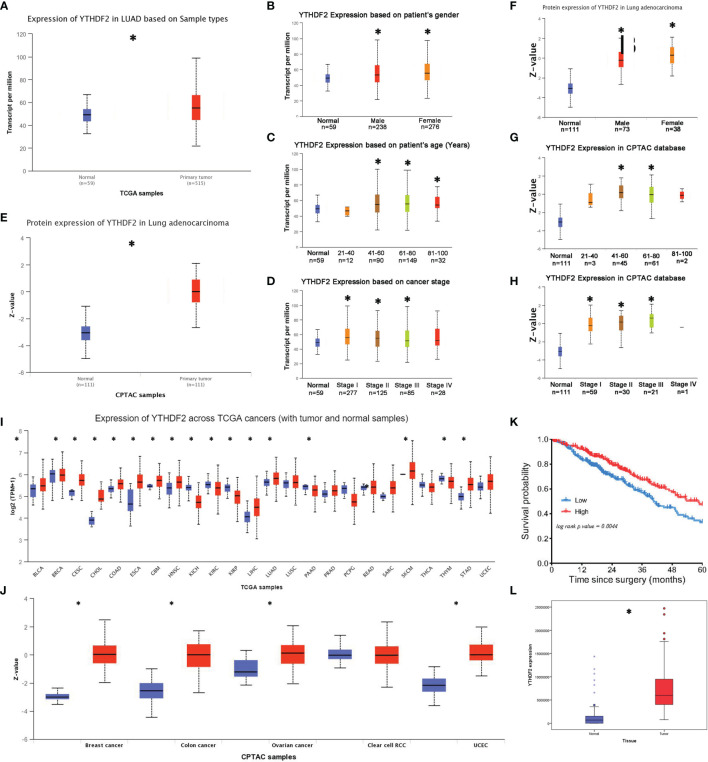
YTHDF2 expression in lung adenocarcinoma. **(A)** YTHDF2 expression in lung adenocarcinoma tissues and normal tissues in TCGA database. **(B–D)** YTHDF2 expression in different patient subgroups compared with normal controls in TCGA cohort. **(E)** YTHDF2 expression in lung adenocarcinoma tissues and normal tissues in the CPTAC database. **(F–H)** YTHDF2 expression in different patient subgroups compared with normal controls in the CPTAC cohort. **(I)** Pancancer analysis of YTHDF2 expression in TCGA database. **(J)** Pancancer analysis of YTHDF2 in the CPTAC database. **(K)** Analysis of the OS of patients with lung adenocarcinoma in TCGA database. **(L)** YTHDF2 expression in lung adenocarcinoma tissues and normal tissues in the CNHPP database. *Statistical difference (P < 0.05).

In TCGA database, significantly higher YTHDF2 mRNA levels were observed in lung adenocarcinoma tissues than in normal tissues (**Figure 1A**). A subgroup analysis produced similar results in patients of different sexes, most ages and most tumour-node-metastasis (TNM) stages, except for some patients aged less than 40 years and patients with stage IV tumours (**Figures 1B–D**). In the CPTAC database, the YTHDF2 expression level was substantially increased in tumour tissues (**Figure 1E**), and the subgroup analysis revealed that this abnormal expression pattern was observed in most patients, except for some patients aged less than 40 years and greater than 80 years; in addition, the increase was not significant in patients with stage IV tumours (**Figures 1F–H**).

The pancancer analysis showed abnormally increased YTHDF2 expression in many tumour tissue types, such as head and neck squamous cell carcinoma, bladder urothelial carcinoma, cervical squamous cell carcinoma, cholangiocarcinoma, oesophageal carcinoma, stomach adenocarcinoma and lung squamous cell carcinoma, in TCGA database (**Figure 1I**). Similarly, increased YTHDF2 expression was observed in many different tumour tissues, such as breast carcinoma, colon carcinoma, ovarian carcinoma and uterine corpus endometrial carcinoma, in the CPTAC database (**Figure 1J**).

A Kaplan–Meier survival analysis was performed for TCGA cohort to explore the relationship between YTHDF2 expression and survival outcomes of patients with lung adenocarcinoma. The results revealed that patients with lower YTHDF2 expression had shorter overall survival (OS) times (**Figure 1K**), and this trend was not as apparent in other lung cancer types, such as small cell lung cancer or squamous cell lung cancer.

A proteomic analysis was performed for 103 patients in the CPTAC cohort to verify the differential YTHDF2 expression in lung adenocarcinoma in the Asian population. The results showed increased expression in tumour tissues compared with normal tissues (**Figure 1L**).

### YTHDF2 Knockdown Inhibits the Proliferation and Colony Formation of Lung Adenocarcinoma Cells

A549 and H1299 cell lines were selected for our study to explore the effect of YTHDF2 on lung adenocarcinoma cells. YTHDF2 expression was detected in A549 and H1299 cell lines, and a small interfering RNA was designed to knock down YTHDF2 expression. YTHDF2 expression was effectively knocked down by 3 small interfering RNAs (siRNAs), and then the second siRNA was selected for subsequent experiments to control the stability.

**Figure 2:**
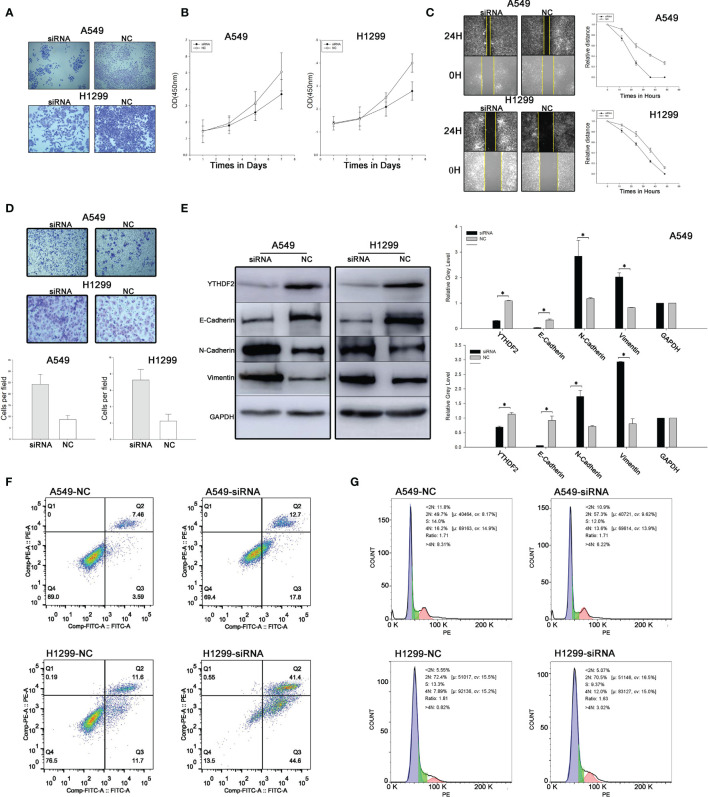
YTHDF2 knockdown leads to increased migration and invasion and decreased proliferation of lung adenocarcinoma cell lines. **(A)** Colony formation assay using normal and YTHDF2 knockdown cell lines. **(B)** MTT assay using normal and YTHDF2 knockdown cell lines. **(C)** Wound healing assay using normal and YTHDF2 knockdown cell lines. **(D)** Transwell assay of normal and YTHDF2 knockdown cell lines. **(E)** EMT-related protein expression in normal and YTHDF2 knockdown cell lines was analysed using WB and the relative greyscale values of 3 repetitions are shown. **(F)** Apoptosis status in normal and YTHDF2 knockdown cell lines was analysed using flow cytometry. **(G)** Cell cycle status in normal and YTHDF2 knockdown cell lines was analysed using flow cytometry. *Statistical difference (P < 0.05).

A colony formation assay was used to evaluate cell proliferation. Compared with the NC group, the number of tumour cell colonies formed in the siRNA group was much lower. The results indicated obviously suppressed proliferation of cells expressing YTHDF2. An MTT assay was used to verify the change in proliferation, and a similar result was obtained (**Figures 2A, B**).

### YTHDF2 Knockdown Promotes the Migration and Invasion of Lung Adenocarcinoma Cells

Wound healing assays and Transwell assays were performed to evaluate cell migration and invasion. Compared with the NC group, the speed of wound healing and the number of invasive cells were noticeably increased, indicating that the migration and invasion of A549 cells were increased when YTHDF2 expression was knocked down (**Figures 2C, D**).

### The EMT Process Was Regulated by YTHDF2 Knockdown

Levels of EMT-related proteins, including E-cadherin, N-cadherin and vimentin, were analysed using WB. The levels of epithelial markers, such as E-cadherin, were reduced, while the levels of mesenchymal markers, such as vimentin and N-cadherin, were substantially increased when YTHDF2 was knocked down (**Figure 2E**).

### YTHDF2 Knockdown Promotes the Apoptosis of Lung Adenocarcinoma Cells

Annexin V FITC/PI double-label staining was used to detect apoptosis, and the results showed that the proportion of apoptotic cells increased significantly after YTHDF2 knockdown compared with normal cells in both the A549 cell line and H1299 cell line (**Figure 2F**). However, in cell cycle experiments, no significant difference was observed between normal and YTHDF2 knockdown cell lines (**Figure 2G**).

### FAM83D Is a Downstream Target of YTHDF2

**Figure 3:**
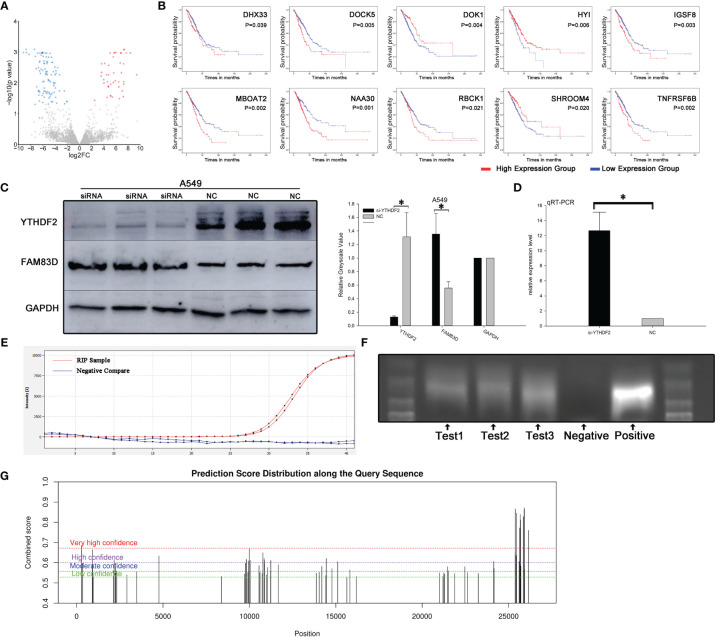
FAM83D was one of the molecules downstream of YTHDF2. **(A)** Volcano map of proteins in pathways downstream of YTHDF2. **(B)** Expression of eleven survival-related proteins in pathways downstream of YTHDF2 in lung adenocarcinoma. **(C)** FAM83D protein expression level after YTHDF2 knockdown, as measured using WB. **(D)** FAM83D mRNA levels after YTHDF2 knockdown, as measured using qRT–PCR. **(E)** RIP assay showing that the FAM83D mRNA was enriched by YTHDF2 antibody-bound magnetic beads, and actin was used as a negative control. **(F)** Bands of three independent RIP assays analysed using DNA agarose gel electrophoresis. **(G)** Prediction of m6A binding sites in FAM83D mRNA. *Statistical difference (P < 0.05).

A proteomics analysis was performed three times independently between YTHDF2 knockdown and normal A549 cells to explore potential downstream pathways of YTHDF2. One hundred forty-two differentially expressed proteins were identified (**Figure 3A**), and further analysis revealed 11 proteins associated with the OS of patients with lung adenocarcinoma: DHX33, DOCK5, DOK1, FAM83D, HYI, IGSF8, MBOAT2, NAA30, RBCK1, SHROOM4 and TNFRSF6B (**Figure 3B**).

**Figure 4:**
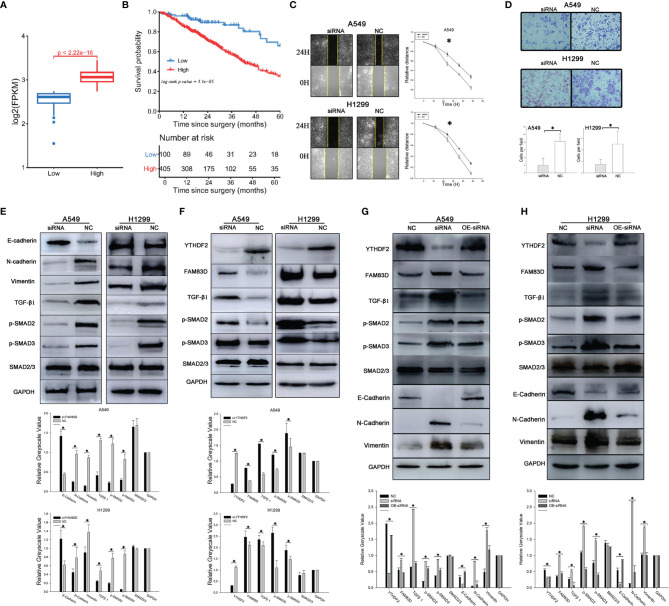
YTHDF2 negatively regulates the FAM83D-TGFβ1-pSMAD2/3 pathway. **(A)** FAM83D expression in different patient subgroups compared with normal controls in TCGA cohort. **(B)** Analysis of OS of patients with lung adenocarcinoma from different FAM83D expression groups in TCGA cohort. **(C)** Wound healing assay using normal and FAM83D knockdown cell lines. **(D)** Transwell assay using normal and FAM83D knockdown cell lines. **(E)** Levels of TGFβ1-pSMAD2/3 signalling proteins and EMT pathway components in normal and FAM83D knockdown cell lines were measured using WB, and the relative greyscale values of 3 repetitions are shown. **(F)** Levels of TGFβ1-pSMAD2/3 signalling proteins in normal and YTHDF2 knockdown cell lines were measured using WB, and the relative greyscale values of 3 repetitions are shown. **(G, H)** The rescue experiment after YTHDF2 expression was knock down in A549 and 1299 cell lines. NC, normal compare; siRNA, knockdown group; OE-siRNA, overexpression by plasmid after knockdown by siRNA group. *Statistical difference (P < 0.05).

Based on a literature review, FAM83D, an upregulated protein in YTHDF2 knockdown cells, attracted our attention (**Figures 4A, B**). Proteomics analysis showed that FAM83D expression increased significantly when YTHDF2 expression was knocked down. RT–PCR showed that FAM83D mRNA expression was significantly increased in YTHDF2 knockdown A549 cells compared with normal A549 cells, and a similar change in the protein level was observed using WB (**Figures 3C, D**).

RIP was performed with YTHDF2 antibody-bound Protein A/G magnetic beads, which captured the YTHDF2 protein and downstream target mRNA, to verify the effect of YTHDF2 on FAM83D mRNA expression. After co-immunoprecipitation, the FAM83D mRNA was detected using qRT–PCR. Actin was used as a negative control and was not detected after RT–PCR amplification (**Figure 3E**). Agarose gel electrophoresis of PCR products was performed to verify the results of three independent RIP assays. Bands similar to the positive control were observed (**Figure 3F**). What’s more, further analysis showed that there were multiple potential m6A binding sites on the mRNA of FAM83D (**Figure 3G**)

### FAM83D Knockdown Inhibits the Migration and Invasion of Lung Adenocarcinoma Cells *via* TGFβ1-pSMAD2/3 Signalling

We aimed to explore the function and mechanism of FAM83D in lung adenocarcinoma. Thus, an siRNA targeting FAM83D was designed and synthesized, and WB indicated that the siRNA efficiently silenced FAM83D expression within7-9 days. Results of cell-based assays showed significantly reduced invasion and migration of FAM83D knockdown A549 cells compared with normal A549 cells (**Figures 4C, D**).

The levels of molecules downstream of FAM83D changed significantly after FAM83D silencing. TGFβ1 and pSMAD2 levels were substantially decreased after FAM83D expression was knocked down, while little change was observed in total SMAD2 expression (**Figure 4E**). When we knocked down YTHDF2 expression, the opposite effect was observed: FAM83D was upregulated, and TGFβ1, pSMAD2 and pSMAD3 levels increased, but little change was observed in total SMAD2/3 expression (**Figure 4F**).

A YTHDF2 overexpression plasmid was transfected into the two YTHDF2 knockdown cell lines for the rescue experiment to further confirm the effect of YTHDF2 on FAM83D and downstream pathways. YTHDF2 expression increased in cells transfected with the plasmid, and FAM83D expression was decreased. Subsequent experiments confirmed decreased levels of TGFβ1 and pSMAD2/3, while total SMAD2/3 levels changed little after transfection. The overexpression plasmid reversed the changes in the FAM83D-TGFβ1-pSMAD2/3 pathway caused by YTHDF2 knockdown (**Figures 4G, H**).

## Discussion

Cancer metastasis is responsible for more than 90% of cancer-associated deaths (20). With the development of tumours, the migration and invasion abilities of cancer cells are enhanced, which is why tumour metastasis occurs. The process by which tumour cells acquire invasion and migration abilities is regulated by many factors. As an important posttranslational modification, RNA methylation, such as m6A methylation, has been proven to be an important factor in cancer metastasis. However, the mechanism by which the m6A modification regulates the metastasis of lung adenocarcinoma remains unclear. In this study, we focused on the role of YTHDF2, a m6A modification reader, in the invasion and migration of lung adenocarcinoma cells.

Our study revealed abnormally high YTHDF2 expression in tumour tissues. Moreover, higher YTHDF2 expression was associated with prolonged OS, indicating that YTHDF2 may function as an inhibitory factor in lung adenocarcinoma. Further studies are needed to determine whether the abnormally high expression of YTHDF2 indicates an oncological role for this protein. As an important m6A modification reader, YTHDF2 has been confirmed in the literature to recognize target mRNAs and promote their degradation; thus, the expression of downstream proteins is inhibited. The literature also shows very different roles for YTHDF2 in different tumour types. In hepatocellular carcinoma, some scholars postulate that YTHDF2 promotes cancer development (11); however, others scholars observed that YTHDF2 suppresses cell proliferation and growth (12). Interestingly, in pancreatic cancer cells, a phenomenon called the “migration-proliferation dichotomy” has been observed: in this case, YTHDF2 promotes proliferation but inhibits migration and invasion (21). In lung adenocarcinoma, few reports have described the role of YTHDF2 in tumorigenesis and development.

The aforementioned results showed reduced proliferation of cells after YTHDF2 knockdown, while the invasion and migration abilities increased. We speculate that YTHDF2 may regulate cell proliferation, invasion and migration through two different mechanisms. Flow cytometry was used to explore the changes in apoptosis and cell cycle progression as a method to further explore the regulatory effect of YTHDF2 on cell proliferation. The proportion of apoptotic cells increased significantly after YTHDF2 knockdown. Therefore, we propose that YTHDF2 may regulate cell proliferation through apoptosis-related pathways.

Subsequently, YTHDF2 expression was knocked down in lung adenocarcinoma cells, and increased invasion and migration was observed. Because the migration and invasion of cells are closely related to the metastasis and progression of malignant disease, we speculate that YTHDF2 may play an important role in tumour metastasis. Several previous studies have shown that in the process of cell migration and invasion, various molecular biological characteristics change significantly, and the most obvious changes are related to the EMT. Generally, the EMT plays a key role in tumour development and metastasis. During the EMT process, cancer cells lose their polarity and gain mesenchymal characteristics, which increases their adhesion ability (22). At the protein level, the EMT is typically characterized by the reduction or loss of epithelial markers, such as E-cadherin, and an increase in the levels of mesenchymal markers, such as N-cadherin and vimentin (23). In our study, a decrease in E-cadherin expression and an increase in N-cadherin and vimentin expression were observed after siRNA transfection, suggesting that the EMT was promoted after YTHDF2 silencing. At the same time, the invasion and migration abilities of tumour cells increased. The results indicated that YTHDF2, which is upstream of the EMT pathway, suppresses cell migration and invasion by inhibiting the EMT process in lung adenocarcinoma.

YTHDF2 affected the migration and invasion of tumour cells by regulating the EMT, but the pathological pathway by which YTHDF2 induces the EMT remained unclear. A proteomics analysis was applied to identify differentially expressed proteins and explore possible pathological pathways; this analysis revealed 142 differentially expressed proteins. The differential expression of these proteins may be due to the knockdown of YTHDF2 expression; therefore, we focused on identifying proteins downstream of YTHDF2. Among the candidate proteins, a series of proteins associated with the prognosis of patients with lung adenocarcinoma were identified, and further assessed by examining the literature, a differentially expressed protein called FAM83D, the mRNA of which was predicted to contain multiple m6A binding sites, was selected for subsequent analysis.

FAM83D is a FAM protein, and 8 members of this protein family have been identified. Recent studies have revealed that most of these members are closely related to the occurrence and development of various types of cancer. As reported by Wang G et al, FAM83A has been confirmed to be highly expressed in lung adenocarcinoma and is associated with a poor prognosis (24). FAM83B inhibits cisplatin resistance in ovarian cancer by inhibiting the Wnt pathway (25), and high FAM83F expression plays a pro-oncogenic role in papillary thyroid cancer (26). The role of FAM83D in the occurrence and development of malignant tumours has also been preliminarily studied; FAM83A, FAM83C, FAM83D and FAM83E promote human mammary epithelial cell (HMEC) transformation (27). FAM83D is associated with tumorigenesis and gemcitabine resistance in pancreatic adenocarcinoma (28) and might be a potential tumour biomarker in triple-negative breast cancer (29). However, few studies examining FAM83D in lung adenocarcinoma have been published.

In this study, a RIP assay was conducted to verify the regulatory effect of YTHDF2 on the FAM83D mRNA. In this assay, Protein A/G magnetic beads were used to bind the antibody, the bound antibody specifically captured the target protein, and the protein bound to the downstream target mRNA. Then the mRNA was identified by subsequently performing RT–PCR. Actin was used as an internal reference that should not be enriched in YTHDF2 antibody-bound magnetic beads to avoid false-positive results. The results confirmed that FAM83D mRNA was enriched on YTHDF2 antibody-bound magnetic beads, indicating that YTHDF2 exerted a direct effect on FAM83D mRNA and that it might be an upstream regulator of FAM83D. Next, WB and qRT–PCR were performed in the YTHDF2 knockdown A549 cell line. The results showed a significant increase in FAM83D mRNA and protein levels, which indicated the inhibitory effect of YTHDF2 on the FAM83D mRNA. As an m6A modification reader, YTHDF2 inhibits target protein expression by recognizing the target mRNA and promoting its degradation. Cytological experiments verified the biological effects and explore the oncological function of the YTHDF2-FAM83D pathway.

Subsequently, siRNA was transfected into cell lines in order to knock down the FAM83D expression. Most tumour cells did not survive normally after FAM83D was knocked down (data not shown in this article), indicating that FAM83D might be a crucial protein for the survival of tumour cells. The concentration of the siRNA we used was reduced in the follow-up study. The viability of the cells decreased significantly, and the invasion and migration of tumour cells were obviously inhibited after transfection, indicating that FAM83D might be involved in the regulation of tumour cell migration and invasion.

In the next study, we explored the downstream pathway of FAM83D, and the qRT–PCR and WB results showed that FAM83D or YTHDF2 knockdown caused abnormal expression of TGFβ1-SMAD2/3 pathway components. The TGFβ1-pSMAD2/3 pathway is one of the most important pathological pathways involved in the development of malignant tumours (30). Changes in the TGFβ1-pSMAD2/3 pathway significantly can affect the proliferation, migration and invasion of tumour cells (31). Moreover, the TGFβ1-pSMAD2/3 pathway significantly regulates the EMT of tumour cells. When FAM83D was knocked down with an siRNA, the levels of TGFβ1, pSMAD2 and pSMAD3 were significantly decreased, while little change in the expression levels of total SMAD2 and total SMAD3 was observed. When YTHDF2 was knocked down by an siRNA, the levels of FAM83D, TGFβ1, pSMAD2 and pSMAD3 were significantly increased, while little change was observed in the levels of total SMAD2 and total SMAD3. In the subsequent rescue experiment, the overexpression plasmid was transfected into YTHDF2 knockdown cells, the expression of YTHDF2 was restored, and the changes in the FAM83D-TGFβ1-pSMAD2/3 pathway were reversed. Therefore, FAM83D-TGFβ1-pSMAD2/3 is one of the pathological pathways downstream of YTHDF2.

## Conclusions

We focused on the abnormal expression of an m6A reader called YTHDF2 in lung adenocarcinoma and proved that YTHDF2 could inhibit the migration and invasion of lung adenocarcinoma cells by regulating the FAM83D-TGFβ1-pSMAD2/3 pathway. This study helps us understand the pathological changes involved in the metastasis of lung adenocarcinoma, and may provide a potential target for the management of advanced lung adenocarcinoma.

## Data Availability Statement

Some results in this study are based upon data generated by TCGA Research Network: [https://www.cancer.gov/tcga]. The data used in this study can be acquired for free via: UCSC XENA browser [https://xenabrowser.net/], The Human Protein Atlas database [https://www.proteinatlas.org/], UALCAN browser [http://ualcan.path.uab.edu/analysis.html], SRAMP prediction server [http://www.cuilab.cn/sramp/]. LC–MS/MS data were uploaded in PRIDE database: Private Project PXD0299392.

## Author Contributions

TZ has designed the study, finished the experiment and drafted the manuscript. MW has designed the study and analyzed the data. XZ, KS, and KQ participated in the analysis of data and the interpretation of results. SW participated in proteomic analysis. WY and YG played an important role in the conception of our study. XQ and YZ revised the manuscript and decided to submit the manuscript. All authors contributed to the article and approved the submitted version.

## Funding

This work was supported by the Beijing Postdoctoral Research Foundation (No. 2021-ZZ-003) and the National Key Program for Basic Research of China (2020YFE0202200).

## Conflict of Interest

The authors declare that the research was conducted in the absence of any commercial or financial relationships that could be construed as a potential conflict of interest.

## Publisher’s Note

All claims expressed in this article are solely those of the authors and do not necessarily represent those of their affiliated organizations, or those of the publisher, the editors and the reviewers. Any product that may be evaluated in this article, or claim that may be made by its manufacturer, is not guaranteed or endorsed by the publisher.
