# Supplementary material for: YTHDF2 Inhibits the Migration and Invasion of Lung Adenocarcinoma by Negatively Regulating the FAM83D-TGFβ1-SMAD2/3 Pathway
Source: Front Oncol. 2022 Feb 2;12:763341. doi: 10.3389/fonc.2022.763341 (PMC8847186; doi:10.3389/fonc.2022.763341)
Supplement: Supplementary file 1 [file DataSheet_1.pdf]

Antibody

Primary antibody

Number	Protein name	brand and Catalog number	Dilution
1	anti-YTHDF2	Proteintech 24744-1	1:2000
2	anti-FAM83D	Abcam ab236882	1:3000
3	anti-E-Cadherin	Cell Signaling Technology 14472	1:1000
4	anti-N-Cadherin	Cell Signaling Technology 13116	1:1500
5	anti-Vimentin	Proteintech 10366-1	1:3000
6	anti-TGFβ1	Immunoway YT1640	1:2000
7	anti-phospho SMAD2	Abcam ab188334	1:5000
8	anti-phospho SMAD3	Abcam ab52903	1:2000
9	anti-SMAD2/3	santa cruz sc-133098	1:1000
10	GAPDH	Proteintech 60004-1	1:30000

Secondary Antibody

Number	Protein name	brand	Dilution	
1	HRP-conjugated Goat Anti-Mouse	Proteintech SA00001-1	1:3000	
2	HRP-conjugated Goat Anti-Rabbit	Proteintech SA00001-2	1:4000	

Primer Sequence

	gene name	Primer sequence 5'-3'		
	FAM83D-Forward1	AACCACTGACTTCCACAATCCT		
	FAM83D-Reverse1	CAAAACAAACCCCTGTATCCAT		
	FAM83D-Forward2	ACGTTGATTGATGGCATCCG		
	FAM83D-Reverse2	CCTTGGACTGTGGTTTTCGG		

SiRNA Sequence

		SiRNA sequence 5'-3'		
YTHDF2	siRNA1	5' -GCCCAAUAAUGCAUAUACUTT-3'		
	siRNA2	5' -GCUCUGGAUAUAGUAGCAATT-3'		
	siRNA3	5' -TTGGCTATTGGGAACGTCCTT-3'		
	siRNA4	5' -AAGGACGTTCCCAATAGCCAA-3'		
FAM83D	siRNA1	5' - GCAGUAACUUGGUAAUUCUTT-3'		
	siRNA2	5' - CGGACUAUCACAGGAAAUATT-3'		
	siRNA3	5' -CCUCUACUGUUAGUGAGGAdTdT-3'		
	siRNA4	5' -GCAGUCUCAUAAGAUUAUATT-3'		
